# Prevention of White Spot Lesions Induced by Fixed Orthodontic Therapy: A Literature Review

**DOI:** 10.3390/dj13030103

**Published:** 2025-02-27

**Authors:** Francesco Saverio Ludovichetti, Edoardo Stellini, Andrea Zuccon, Patrizia Lucchi, Niccolò Dessupoiu, Sergio Mazzoleni, Roberta Gaia Parcianello

**Affiliations:** 1 Dentistry Section, Department of Neurosciences, Università degli Studi di Padova, 35122 Padova, Italy; edoardo.stellini@unipd.it (E.S.); andrea.zuccon@unipd.it (A.Z.); patrizia.lucchi@unipd.it (P.L.); niccolo.dessupoiu@studenti.unipd.it (N.D.); sergio.mazzoleni@unipd.it (S.M.); 2 Department of Biomedical, Surgical and Dental Sciences, University of Milan, 20122 Milan, Italy; roberta.parcianello@unimi.it

**Keywords:** white spot lesions, fixed orthodontic treatment, enamel demineralization, preventive strategies

## Abstract

**Objectives**: This study aims to review the scientific literature on the prevention of white spot lesions (WSLs) associated with fixed orthodontic treatment. WSLs result from enamel demineralization and pose aesthetic and functional challenges. The study evaluates the effectiveness of various preventive interventions to reduce the risk of WSLs during orthodontic care. **Methods**: A comprehensive literature search was conducted using MeSH terms such as “white spot”, “fixed orthodontic treatment”, “CPP-ACP”, “topical fluoride”, and “demineralized lesion”, combined with the Boolean operator ‘AND’. Databases searched included PubMed, EMBASE, Scopus, and OpenGrey, focusing on studies published between January 2014 and September 2024. The inclusion criteria required studies to evaluate interventions aimed at reducing WSL risk in patients undergoing fixed orthodontic treatment. A total of 41 articles were screened, with 17 selected based on relevance and methodological rigor. **Results**: The review identified several effective strategies for preventing WSLs. Topical fluoride applications, particularly high-concentration varnishes, significantly reduced WSL prevalence, with some studies favoring fluoride varnish over other interventions. CPP-ACP and CPP-ACPF formulations demonstrated potential for remineralizing demineralized enamel, especially when combined with fluoride toothpaste. High-fluoride toothpaste and acidulated phosphate fluoride mouthwash were effective in reducing lesion incidence, emphasizing the importance of patient compliance in daily oral hygiene routines. Professional interventions, such as fluoride varnish applications, showed enhanced outcomes when re-applied periodically. **Conclusions**: Preventing WSLs during orthodontic treatment is crucial for ensuring the aesthetic and functional success of therapy. Strategies combining fluoride-based interventions with casein phosphopeptide formulations offer significant benefits. However, patient education and adherence to recommended oral hygiene practices remain critical. Further research is needed to compare the long-term effectiveness of these interventions and to explore new technologies for WSL prevention.

## 1. Introduction

Dental caries is a chronic, multifactorial infectious disease caused by bacteria, impacting a significant portion of the global population, especially school-aged children and adults. It develops when cariogenic bacteria in dental plaque metabolize sugars, resulting in tooth demineralization and cavity formation. The disease is influenced by factors such as sugar consumption, inadequate oral hygiene, and individual host conditions [1]. White spot lesions (WSLs) are early indicators of caries, caused by enamel demineralization without cavity formation, resulting in a chalky appearance. Remineralization can reverse this process, but continued demineralization may lead to irreversible cavities. A balance between demineralization and remineralization is crucial, though cosmetic scars from WSLs may remain [2]. WSLs are common in orthodontic patients, often appearing within weeks of treatment, particularly around brackets or under molar bands. Fixed orthodontic appliances complicate oral hygiene, leading to plaque buildup and WSL formation, which can hinder treatment success and raise aesthetic concerns [3]. Orthodontic brackets are crucial parts of fixed orthodontic appliances and contribute significantly to enamel demineralization during extended orthodontic treatment. Their intricate design makes it difficult to maintain proper oral hygiene, restricting effective cleaning around the brackets and providing extra surfaces for plaque buildup [4]. Orthodontic materials with rougher surfaces provide a greater area for bacterial adhesion and help protect the biofilm from shear forces. Additionally, materials with higher surface free energy thermodynamically promote bacterial adhesion and biofilm formation. Along with the material, the shape and size of the brackets also influence bacterial adhesion and biofilm development. As a result, smaller and simpler bracket designs are preferred to reduce bacterial adhesion and plaque accumulation [5]. Therefore, patients receiving orthodontic treatment need education on proper oral hygiene practices, emphasizing the importance of achieving good oral hygiene prior to starting treatment and recognizing the challenges posed by orthodontic appliances [6]. Orthodontic patients are particularly at risk for white spot lesions, which require preventive measures such as remineralizing agents and fluoride treatments. These lesions, along with enamel demineralization, are common issues during orthodontic treatment with fixed appliances. Patients with malocclusion often struggle to maintain proper oral hygiene due to hard-to-reach areas, leading to plaque buildup. White spot lesions typically appear on the upper lateral incisors and canines around the brackets within a month, though full cavity formation usually takes at least six months [7]. Key risk factors for dental caries include high sugar intake, poor oral hygiene, lack of fluoride exposure, and infrequent dental visits, with children and adolescents particularly affected. Socioeconomic and demographic factors such as occupation, education, income, gender, age, and ethnicity also contribute to caries vulnerability. Regular brushing with fluoride toothpaste and routine dental checkups are essential for preventing caries and maintaining oral health [8]. Wearing a fixed orthodontic appliance can cause discomfort and increase plaque buildup. White spot lesions may form on the teeth during long-term orthodontic treatment, affecting their appearance after occlusion correction [9]. Preventive strategies to reduce cavity risk include systemic fluoride supplements, artificial water fluoridation, and the use of fluoride toothpaste. The optimal fluoride concentration in fluoridated water is 1 ppm, balancing anticariogenic benefits while minimizing the risk of dental fluorosis. In patients with molar incisor hypomineralization (MIH), amelogenesis imperfecta, or fluorosis, fixed appliances may not be recommended due to issues with adhesion, enamel fragility during orthodontic treatment, and an increased risk of plaque accumulation. The porosity of the dental surface, combined with the challenges of cleaning while wearing braces, can exacerbate these problems [10]. CPP-ACP (Casein Phosphopeptide-Amorphous Calcium Phosphate) releases calcium and phosphate ions, which are essential for remineralizing tooth enamel. It helps repair early enamel damage, such as white spot lesions, by delivering these ions to demineralized areas. Additionally, it prevents further demineralization by protecting enamel from acid attacks. The compound is commonly used in oral care products to strengthen and restore enamel. In essence, CPP-ACP supports tooth health by promoting remineralization and preventing decay [11]. For what concerns fluoride, instead, it helps remineralize teeth by replacing hydroxide ions in the enamel with fluoride, forming fluorapatite, which is more resistant to acid attacks. This process restores lost minerals and strengthens the enamel, making it more resistant to cavities and decay [12].

The aim of the study is to review the existing literature to evaluate the effectiveness of different preventive strategies for reducing the risk of white spot lesions in patients undergoing fixed orthodontic treatment.

## 2. Materials and Methods

For the preparation of this work, a comprehensive literature review was conducted, focusing on articles that evaluated the effectiveness of various active ingredients and preventive interventions aimed at reducing the risk of white spot lesions induced by fixed orthodontic therapy.

The reviewers worked in pairs (RGP and FSL), identifying work that met the following inclusion criteria: clinical cases, randomized clinical trials or systematic reviews, studies performed on subjects who are undergoing orthodontic fixed therapy; and studies regarding WSL prophylaxis in subjects who are in orthodontic therapy.

Exclusion criteria were (1) studies involving therapy of WS after orthodontic therapy; (2) studies involving cure of WS unrelated to orthodontics; (3) in vitro studies; (4) animal studies; (5) systematic reviews, narrative reviews, and meta-analyses.

The Medical Subject Headings (MeSH) terms used in the search strategy included “Dental Caries”, “Orthodontic Appliances”, “Tooth Demineralization”, “Fluorides”, and “Caseins.” Additionally, relevant non-MeSH keywords such as “White Spot Lesions”, “Fixed Orthodontic Treatment”, “CPP-ACP”, and “Topical Fluoride” were incorporated to broaden the search and ensure the inclusion of studies not indexed under MeSH terminology. The search was conducted in the PubMed, EMBASE, Scopus, and OpenGrey databases, covering literature published between January 2014 and September 2024 (Appendix A). The screening took place on the 16th of June 2024 and the selection process took place between June and September 2024.

**Figure 1:**
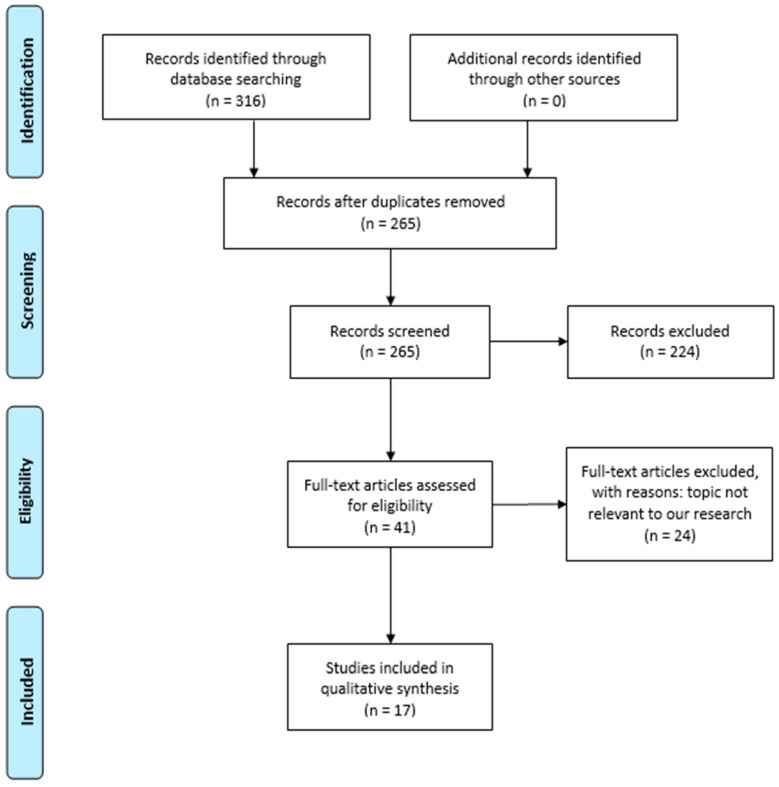
PRISMA 2009 flow diagram.

The identified articles were first screened based on their title and abstract, resulting in 41 eligible articles. Subsequently, full-text articles were reviewed, and a total of 17 articles were selected for inclusion in this thesis. These included reviews, systematic reviews, in vitro studies, in vivo studies, and randomized controlled trials (Figure 1).

## 3. Results

Based on the research conducted in the relevant database, 41 potentially relevant articles were selected. Subsequently, full-text articles were reviewed, according to the inclusion and exclusion criteria, 17 articles were chosen for this work, including randomized clinical trials, systematic reviews and meta-analyses, in vivo studies, and in vitro studies. The following table provides a concise and schematic summary of the selected studies, based on the international scientific literature. Organized in columns, the table summarizes the title of the selected scientific article according to the inclusion and exclusion criteria, the author, the year of publication, the study objective, the materials and methods used, and the conclusions reached.

These included reviews, systematic reviews, in vitro studies, in vivo studies, and randomized controlled trials (Table 1).

The 2016 study by Lopatiene et al. is a systematic review aimed at updating evidence on preventing white spot lesions (WSLs) using fluoride-containing materials and/or casein phosphopeptide-amorphous calcium phosphate (CPP-ACP) during and after fixed orthodontic treatment. Twelve studies were included, covering clinical trials, cohort studies, and case reports. Four studies showed no significant improvement in WSLs with fluoride products (varnish, toothpaste), while four others showed significant improvement with fluoride-based materials. The use of CPP-ACP was found to be more effective than fluoride rinses for post-orthodontic remineralization. Overall, nine of the twelve studies indicated that treatments with fluoride and/or CPP-ACP are effective in managing and preventing WSLs [13].

The 2019 study by Benson et al. examined the effectiveness of topical fluoride in preventing white spot lesions (WSLs) in orthodontic patients. It found that fluoride varnish was the most effective treatment, significantly reducing new WSLs (11.7%) compared to a placebo group (29.7%). Lower concentrations of fluoride varnish were less effective. Fluoride gel applied every three months showed no significant difference in WSL development, suggesting less frequent applications are less effective. Professional fluoride foam, applied every two months, also reduced new lesions, emphasizing the importance of frequent fluoride use. High-fluoride toothpaste (5000 ppm) provided some protection, with combinations of amine or stannous fluoride outperforming standard sodium fluoride. Overall, professional fluoride varnishes, particularly those with higher fluoride concentrations, were the most effective method, while frequency and concentration were key for optimal results [14].

The 2023 study by Sonesson et al. analyzed the preventive effects of regular fluoride varnish application during orthodontic treatment, focusing on its ability to prevent white spot lesions (WSLs). The study reviewed seven randomized controlled clinical trials with a minimum 12-month duration, where fluoride varnish was applied at least every three months around the brackets. The varnishes tested included 5% sodium fluoride, 5% sodium fluoride with CPP-ACP, difluorosilane, and 1.5% ammonium fluoride. The frequency of applications ranged from every 4 weeks to every 3 months, and study durations ranged from 12 to 26 months. Results showed that regular fluoride varnish application significantly reduced the incidence of WSLs, with most studies indicating a reduction in enamel demineralization. The combination of fluoride with other substances, like CPP-ACP, provided additional benefits for remineralization. Overall, regular fluoride varnish application was effective in preventing WSLs during orthodontic treatment, with application frequency being a key factor in treatment success [15].

The 2016 study by Perrini et al. aimed to evaluate the in vivo effectiveness of Duraphat fluoride varnish in preventing white spot lesions in orthodontic patients. A split-mouth design was used with 24 participants, where fluoride varnish was applied to quadrants 1 and 3, and quadrants 2 and 4 served as controls. Participants were randomly assigned to receive Duraphat every three months (Group 1) or every six months (Group 2). The 5% sodium fluoride varnish was applied around the brackets, and patients were instructed to follow oral hygiene guidelines. The results showed a tendency for greater demineralization in untreated teeth, but no significant differences were observed across the four measurement periods (3, 6, 9, and 12 months). At nine months, treated incisors showed significantly less demineralization than untreated incisors. However, the overall differences between treated and untreated teeth were not significant, except for the anterior teeth, and the frequency of application (twice vs. four times a year) did not lead to notable differences in effectiveness [16].

The 2020 study by Sonesson et al. investigated the effectiveness of a new fluoride varnish formulation containing 1.5% ammonium fluoride in preventing white spot lesions in adolescents undergoing fixed orthodontic treatment. Participants used fixed appliances for at least twelve months and received either the test varnish or a placebo varnish every six weeks, following orthodontic check-ups and biofilm removal. The placebo varnish had the same color, taste, and handling properties as the test varnish, except for the ammonium fluoride content. After one minute of drying, patients were instructed to avoid eating or drinking for one hour and brush with fluoride toothpaste (1450 ppm). At the end of treatment, there was no significant difference in the prevalence of mild white spot lesions between the test and placebo groups. However, the test varnish group had fewer severe white spot lesions compared to the placebo group at the time of appliance removal [17].

The 2022 study by Pilli et al. compared the clinical efficacy of a neutral sodium fluoride (NaF) mouthwash versus an acidulated phosphate fluoride (APF) mouthwash in reducing white spot lesions (WSLs) after orthodontic treatment. Ninety participants were randomly assigned to two groups: Group A received a weekly 0.2% NaF mouthwash, and Group B received a daily 0.044% APF mouthwash. Participants rinsed with the mouthwash for one minute after brushing their teeth. Assessments were made at four-week (T1), twelve-week (T2), and twenty-four-week (T3) intervals. The NaF group showed a gradual increase in ICDAS scores, indicating enamel demineralization, from T0 to T3. In contrast, the APF group had increasing ICDAS scores until T2, followed by a decrease at T3. Statistically significant changes in ICDAS scores were observed in the NaF group between all periods except T2 to T3, while in the APF group, significant changes occurred between T2 and T3 [18].

Yazarloo et al. conducted a literature review in 2023, including several randomized controlled trials, to compare various preventive and therapeutic measures for addressing white spot lesions (WSLs) induced by fixed orthodontic treatment. A search was conducted through relevant databases, and twenty-three articles met the required inclusion criteria. The selected articles cover both preventive strategies, such as evaluating the effectiveness of products containing CPP-ACP, as well as varnishes, toothpastes, mouthwashes, adhesive agents, or sealants; and therapeutic strategies, such as examining remineralizing agents, fluoride varnishes, and chlorhexidine mouthwashes [19].

A study by Sardana et al., conducted in 2019, investigated the effect of patient-applied topical fluorides in preventing the onset of white spot lesions (WSLs) caused by traditional fixed orthodontics. A comprehensive search was performed across several databases, from which only three randomized controlled clinical trials were selected for inclusion in the study: two focused on prevention, while the third examined the reversal of post-orthodontic white spots. Both studies that assessed the prevention of enamel lesions measured outcomes at the time of bracket removal. One of these studies found a significantly lower number of WSLs in the group using a 250 ppm fluoride mouth rinse compared to the placebo, while the 5000 ppm sodium fluoride toothpaste was significantly more effective than the 1450 ppm toothpaste [20].

The ability of products containing CPP-ACP and CPP-ACPF to prevent and remineralize white spot lesions (WSLs) was studied by Imani et al. in 2019. Four studies included an experimental group with a CPP-ACP-containing product, while nine studies used a product containing CPP-ACPF, for a total of thirteen relevant articles. Both groups were compared with one or more control groups, such as placebo, fluoride toothpaste control, fluoride varnish, or fluoride mouthwash. The follow-up period ranged from three to thirty-six months. Three studies demonstrated greater efficacy of CPP-ACP in remineralizing WSLs, while another study found no superior effect compared to the other groups. Four studies reported a clinical advantage of CPP-ACPF in the prevention and remineralization of WSLs, while the remaining five studies did not show a significant difference compared to the other groups [21].

The 2023 study by Ravi Kiran et al. evaluated the effectiveness of amine fluoride mouthwash as a preventive measure during fixed orthodontic therapy. Participants were followed for six months, starting at the bonding stage, and were randomly divided into two groups. Group A (control) followed a standard oral hygiene routine using only fluoride toothpaste, while Group B (experimental) followed the same routine but added daily use of a 480 ppm amine fluoride mouthwash. Before the intervention, white spot lesion scores were similar between the groups (*p*-value = 0.068). However, after six months, the scores significantly differed (*p*-value = 0.006). The control group showed a significant increase in lesion scores, while the experimental group experienced a decrease, highlighting the effectiveness of amine fluoride mouthwash in preventing enamel lesions during orthodontic treatment [22].

A review conducted by Babadi Oregani et al. in 2022 examined the preventive effect of fluoride interventions, compared to a placebo, on enamel demineralization. Based on the inclusion criteria, seven relevant studies were selected. In four studies involving fluoride varnish, participants in the experimental groups received fluoride varnish applications at various intervals, ranging from four to twenty times during treatment, while the control group patients received either a placebo varnish or no treatment. Another study used two fluoride varnishes for the experimental group, which were applied only once at the beginning of treatment, while the control group received a placebo varnish. In one article, a fluoride mouthwash was used for the experimental group, and a placebo mouthwash was used for the control group. Lastly, the final study compared the effect of a high-fluoride toothpaste (5000 ppm) with a standard fluoride toothpaste (1450 ppm). The results showed significant differences between the experimental and control groups in all these studies. Specifically, the number or severity of enamel lesions was lower in the fluoride-treated groups. However, in the study where the fluoride varnish was applied only once, no significant effect on white spot lesions was observed compared to the control group [23].

The 2018 clinical trial by Rechmann et al. assessed the effect of a combined fluoride toothpaste regimen with MI Paste Plus (MIPP) and MI Varnish (MIV) on preventing and reversing white spot lesions during orthodontic treatment. Forty participants were randomly assigned to experimental and control groups. The experimental group received MI Varnish (10% CPP-ACP, 5% NaF) quarterly and applied MI Paste Plus (10% CPP-ACP, 0.2% NaF, 900 ppm fluoride) every evening after brushing. The control group used fluoride toothpaste (1100 ppm) and fluoride mouthwash (0.05% NaF). The primary outcome was the enamel demineralization index (EDI), measured at baseline, 3, 6, and 12 months. At baseline, the control group’s average EDI score was 37.7, which slightly increased to 41.3 after 12 months. The experimental group’s baseline score was 42.9, decreasing slightly to 40.2 after 12 months. However, the differences between the groups were not statistically significant [24].

The 2014 study by Sonesson et al. aimed to evaluate the preventive effect of high-fluoride toothpaste on white spot lesions during fixed orthodontic treatment. Adolescents undergoing the straight-wire technique for at least one year were included. The experimental group used toothpaste with 5000 ppm sodium fluoride, while the control group used toothpaste with 1450 ppm sodium fluoride, following the same brushing routine. Standardized toothpaste and toothbrushes were provided at the start and every three months. At baseline, no significant differences were found, but by the end of treatment, the high-fluoride toothpaste group had a significantly lower prevalence of white spot lesions (18.1%) compared to the control group (26.6%). The control group had a higher risk of developing new demineralized lesions, and most of the lesions in both groups were thin-margin lesions. More severe lesions were found in 1.2% of the experimental group and 2.3% of the control group [25].

The 2019 in vitro study by Reddy et al. compared the effectiveness of different fluoride treatments in preventing enamel demineralization around orthodontic brackets. The study involved 100 extracted premolar teeth, divided into five groups: Group 1 received fluoride varnish, Group 2 was exposed to acidulated phosphate fluoride (APF) gel, Group 3 had fluoride toothpaste applied, Group 4 rinsed with sodium fluoride mouthwash, and Group 5 served as the control group. The daily cycle included a 6 h demineralization period at pH 4.3 and a 17 h remineralization period. After 14 days, the fluoride varnish (Group 1), APF gel (Group 2), and fluoride toothpaste (Group 3) groups showed significantly lower demineralization compared to the control group. However, the sodium fluoride mouthwash (Group 4) did not show a statistically significant difference from the control group. The study concluded that fluoride varnish, APF gel, and fluoride toothpaste were effective in preventing enamel demineralization, while sodium fluoride mouthwash was not [26].

The most effective prophylactic strategies for preventing white spot lesions during fixed orthodontic therapy were examined by Patano et al. in 2023. This review included clinical studies or case reports conducted exclusively on human subjects undergoing fixed orthodontic therapy, focusing on the prevention of enamel demineralization in traditional orthodontics. A comprehensive search of relevant databases led to the selection of 16 publications for the review. The findings showed that daily acidic phosphate-based mouthwashes were more effective than weekly sodium fluoride mouthwashes in preventing white spot lesions. Additionally, the regular use of ammonium fluoride varnish significantly reduced the occurrence of white spots, as did the use of casein phosphopeptide-amorphous calcium phosphate (CPP-ACP) and fluoride toothpaste. The study also highlighted the potential of CO2 laser treatment in preventing caries and reducing the incidence of enamel demineralization. Finally, no significant differences were observed between fluoride toothpaste and those containing active oxygen in terms of their role in prevention. In summary, daily acidic phosphate mouthwashes, fluoride varnish, CPP-ACP, and fluoride toothpaste were found to be effective preventive measures for white spot lesions during fixed orthodontic treatment, with CO2 lasers offering additional potential for reducing enamel demineralization [27].

The 2019 study by Kau et al. compared the effectiveness of two toothpastes, Clinpro 5000 (1.1% sodium fluoride) and Clinpro Tooth Crème (0.21% sodium fluoride), as well as MI Paste Plus (containing casein phosphopeptide-amorphous calcium phosphate with fluoride, or CPP-ACPF), in preventing white spot lesions in orthodontic patients. Each of the three groups had 40 participants, who applied the selected product twice a day for two minutes over four months. Enamel Decalcification Index (EDI) was used to assess the development of white spot lesions. The results showed Clinpro 5000 (5000 ppm) significantly reduced enamel decalcification compared to Clinpro Tooth Crème (950 ppm), which showed higher levels of enamel demineralization. MI Paste Plus showed marginal significance with a higher EDI than Clinpro 5000. The study concluded that Clinpro 5000 provided the best protection against enamel demineralization, followed by MI Paste Plus, while Clinpro Tooth Crème was less effective in preventing white spot lesions [28].

The 2019 study by Tahmasbi et al. compared the effectiveness of sodium fluoride (NaF), casein phosphopeptide-amorphous calcium phosphate with fluoride (CPP-ACPF; MI Paste Plus), and a water-based cream Remin Pro (containing hydroxyapatite and fluoride) in preventing enamel demineralization. In total, 4 groups of 14 samples each were treated for 14 days. Group 1 (control) underwent only a pH cycle, Group 2 was treated with NaF (0.05% mouthwash for 5 min), Group 3 received MI Paste Plus (applied for 5 min after artificial saliva), and Group 4 was treated with Remin Pro (applied for 5 min after artificial saliva). After treatment, microhardness testing showed that NaF (Group 2) had the least reduction in microhardness, indicating it was the most effective at preventing enamel demineralization. Remin Pro (Group 4) was the second most effective, followed by MI Paste Plus (Group 3), while the control group showed the least improvement. The study concluded that NaF was the most effective in preventing enamel demineralization, followed by Remin Pro, MI Paste Plus, and the control group [29].

The details of the quality assessment of twenty-three eligible studies included in this systematic review were extracted and an assessment of the risk of bias for included studies was performed. Two studies were assessed to be low, two studies were considered to be moderate, and nineteen studies were classified as high risk of bias (Table 2).

## 4. Discussion

Current scientific literature includes numerous studies on the prevention of white spot lesions (WSLs) associated with traditional fixed orthodontic treatment using brackets. This topic is crucial, as the primary goal of orthodontic treatment is to improve the smile’s aesthetics, and demineralized lesions can compromise treatment outcomes, causing patient dissatisfaction. These lesions are often observed on the labial surfaces of maxillary incisors and have been found in up to 96% of patients undergoing orthodontic treatment [17].

Several studies highlight the effectiveness of fluoride varnish in reducing orthodontically induced WSLs [16,17,18,19,21,25,28,29]. Benson et al. (2019) [14] emphasized that professionally applied “passive” fluoride-releasing interventions, such as fluoride varnish, eliminate the need for patient compliance. These materials deliver fluoride at the critical point where it is most needed. Although many fluoride materials release high fluoride levels initially, the release diminishes quickly, often requiring repeated applications. Studies reviewed by Benson et al. [14] showed that reapplying fluoride varnish every six weeks during orthodontic check-ups did not yield significant reductions in WSLs. Similarly, Perrini et al. [16] assessed fluoride varnish over 12 months, showing that treated teeth experienced less demineralization, with statistically significant improvements observed in anterior teeth.

Sonesson et al. [15] noted that the protective effects of fluoride varnish were more pronounced in advanced lesions, emphasizing the importance of preventing severe WSLs, which often require additional interventions such as resin infiltration or bleaching to maintain aesthetic outcomes. Earlier, Sonesson et al. (2020) [17] demonstrated that regular application of a varnish containing 1.5% ammonium fluoride (7700 ppm fluoride) effectively reduced WSLs.

Clinically significant results were observed, with many white margins remineralizing within three months after bracket removal. Studies by Patano et al. [26] and Yazarloo et al. [19] corroborated these findings, supporting the efficacy of fluoride varnish in reducing WSL incidence. Notably, Reddy et al. [25] found that a varnish combining calcium, fluoride, and phosphate showed superior remineralization potential, attributed to its unique manufacturing process.

This varnish has shown superior results in remineralization compared to others, and this potential has been attributed to its particular production process, which combines beta-tricalcium phosphate and sodium lauryl sulfate to create “functionalized” calcium and free phosphate, thus increasing the ability of calcium to be incorporated into enamel [25]. Finally, the authors Babadi Oregani et al. [23], in 2022, analyzed four varnishes with different concentrations and concluded that the acid resistance of enamel among the groups treated with 22,000 ppm and 10,000 ppm did not differ significantly, while both high-dose groups showed considerably higher acid resistance. Consequently, increasing the fluoride concentration up to a certain level improves the preventive effect, confirming that the use of fluoride varnish reduces the formation of WSLs (white spot lesions) [26].

Other studies have examined casein phosphopeptide-amorphous calcium phosphate (CPP-ACP) and its fluoride-enriched variant (CPP-ACPF) [13,19,21,24,28,29]. Imani et al. [20] highlighted that CPP-ACP stabilizes calcium and phosphate ions on the enamel surface, promoting remineralization, especially when combined with fluoride. Daily use of CPP-ACP with fluoridated toothpaste significantly reduced WSLs within a month. However, some studies, such as Rechmann et al. [24], reported inconsistent results regarding its long-term effectiveness, and others noted that fluoride remains more effective than CPP-ACP alone.

CPP-ACP can promote remineralization, and its combination with fluoride appears to have a synergistic effect. In fact, CPP-ACPF, a variant with added fluoride, has shown a significant effect on the remineralization of white spot lesions (WSLs). Several studies have demonstrated that a remineralizing cream containing CPP-ACP, combined with fluoridated toothpaste, is more effective than a placebo cream. Daily use of CPP-ACP with fluoride toothpaste significantly reduced white spot lesions after one month [21]. The research by Lopatiene et al., from 2016, supports this view, stating that the use of fluoride-based supplements and casein phosphopeptide-amorphous calcium phosphate is significantly effective in reducing demineralization during orthodontic treatment. They also note that CPP-ACPF may offer greater benefits than fluoride mouthwash for post-orthodontic remineralization [13].

The study by Yazarloo et al. (2023) [19] also agrees with this, highlighting that the use of MI Paste Plus, containing CPP-ACP with fluoride, does not provide long-term benefits in the treatment of white spot lesions (WSLs), while short-term application showed positive results. The use of CPP-ACP alone did not show significant improvements in WSLs, but it proved effective when combined with the daily use of fluoride toothpaste [19]. On the other hand, the research conducted by Rechmann et al. in 2018 presents a different view. Although it supports the idea that the addition of CPP-ACP enhances the incorporation of fluoride into the plaque, further promoting enamel remineralization, the results were contradictory. Specifically, the treatments considered included quarterly application of MI Varnish, daily application of MI Paste Plus, and brushing with fluoride toothpaste twice a day. While the control group showed an increase in the EDI score (enamel demineralization index) and the experimental group showed a slight decrease, these differences were not statistically significant. Similarly, no significant differences in ICDAS scores were found between the groups [24].

In agreement with Rechmann et al., the study by Tahmasbi et al. (2019) [29] also states that MI Paste Plus partially inhibited enamel demineralization, but without significant differences compared to the control group. Although it somewhat reduced enamel lesion formation, its effectiveness was lower than that of fluoride [29]. Similarly, the study by Kau et al. (2019) [28] compared the use of Clinpro 5000 toothpaste, Clinpro Crème, and MI Paste Plus in preventing white spot formation during orthodontic treatment. It was observed that MI Paste Plus had a lower effect compared to Clinpro 5000 toothpaste, which demonstrated greater remineralizing potential [28]. The findings emphasize the critical role of fluoride-based interventions in preventing WSLs during fixed orthodontic therapy. The consistent success of high-fluoride varnishes and toothpaste in reducing lesion formation suggests that frequent and controlled fluoride exposure enhances enamel resistance to demineralization. Additionally, the potential synergy between CPP-ACP and fluoride highlights the need for further studies assessing the long-term benefits of combination therapies. While some studies suggest that fluoride mouthwashes and gels provide protective effects, patient compliance remains a key factor influencing efficacy.

This review also analyzed the preventive and remineralizing capabilities of various toothpastes, both high-fluoride and those with different formulations [14,19,20,25,26,27,28]. The study by Benson et al. (2019) highlights that interventions involving the use of fluoride toothpaste, which requires patient self-administration, are effective only if used regularly, with patient compliance playing a crucial role in achieving positive outcomes [14]. Research by Yazarloo et al. (2023) reported that brushing teeth twice a day for two minutes with two different fluoride toothpastes (Clinpro 5000 and Clinpro Tooth Creme) is effective in preventing white spot lesions (WSLs), with results similar to those obtained with MI Paste Plus [19]. This aligns with the findings of Kau et al. (2019), who reported that the group treated with Clinpro 5000 showed the lowest EDI percentage. Therefore, Clinpro 5000 toothpaste showed a slightly superior effect compared to the other two pastes used in the study [28]. Similarly, the study by Patano et al. (2023) [27] agrees with Kau et al.’s findings on the effectiveness of the toothpaste in question. Furthermore, it states that toothpaste with active oxygen is just as effective as fluoride-containing toothpaste [27]. The project carried out by Sonesson et al. (2014) [25] aimed to demonstrate that a high-fluoride toothpaste could reduce the metabolic activity of the biofilm. Indeed, the high-concentration toothpaste acts locally at the interface between the biofilm and the tooth, reducing enamel demineralization and promoting remineralization [25].

Sardana et al. (2019) share the same opinion, having compared toothpastes with high and low fluoride concentrations, demonstrating that a higher fluoride concentration offers better prevention of white spot lesions (WSLs) compared to a lower concentration [20]. Finally, in agreement with the aforementioned studies, the research by Reddy et al. (2019) also highlighted that the group treated with fluoride toothpaste achieved significantly better results than the control group in preventing white spot lesions induced by fixed orthodontic therapy [26]. In addition to analyzing various articles on fluoride varnishes, casein phosphopeptide-amorphous calcium phosphate products, and high-fluoride toothpastes, studies on different types of mouthwashes and their preventive capabilities were also examined [18,19,22,26,29]. The study by Pilli et al. (2022) [18] compared the efficacy of a mouthwash containing acidulated phosphate fluoride (APF) with 0.044% NaF against a mouthwash containing 0.02% sodium fluoride (NaF). During the first month of orthodontic treatment, a significant increase in enamel demineralization and initial caries was observed in both groups, indicating that white spot lesions could not be completely prevented in all patients. This confirmed that the total prevention of WSLs is not possible. However, it was found that daily oral rinsing with APF-based formulations was more effective in preventing WSLs compared to weekly rinsing with neutral sodium fluoride [18].

Similarly, Tahmasbi et al. (2019) [29] compared the efficacy of a sodium fluoride (NaF) mouthwash with MI Paste Plus and Remin Pro in preventing WSLs to identify the most effective agent. The results showed minimal changes in enamel microhardness in the NaF group, indicating that NaF was the most effective at preventing enamel demineralization among the groups studied [29]. A different opinion was presented in the research by Reddy et al. (2019) [26], which evaluated the efficacy of a sodium fluoride mouthwash. This study reported slightly superior results compared to the control group, but the differences did not reach statistical significance [26]. Another article by Ravi Kiran et al. (2023) [22] assessed the effectiveness of a low-dose ammonium fluoride mouthwash in preventing white spot lesions during fixed orthodontic treatment. In patients using the ammonium fluoride mouthwash, WSL scores were significantly reduced compared to those following a standard oral hygiene regimen with only fluoride toothpaste. The results showed a lower prevalence of WSLs in the experimental group compared to the control group [22]. Finally, the research by Sardana et al. (2019) [20] compared the preventive capacity of a mouthwash containing 250 ppm of fluoride (150 ppm NaF and 100 ppm ammonium fluoride) with a placebo mouthwash. The authors noted a significant reduction in WSLs in the group using the fluoride mouthwash compared to the placebo, thus supporting the preventive role of self-applied fluorides in the development of WSLs [20].

The limitations of this study include the inability to access additional databases, such as “Science Direct” and “Web of Science”, due to subscription requirements. Additionally, while “Medline” was checked, the proxy prevented access to it. Another limitation is the significant heterogeneity of the studies, which prevented the possibility of conducting a proper quantitative analysis. Further studies on this topic should be conducted, with consideration given to staging white spots to evaluate their progression over time. Additionally, it would be valuable to explore whether better outcomes result from the application of fluoride, phosphopeptide-amorphous compounds, or other treatments aimed at reducing white spots. The potential of emerging bioactive materials, such as nanohydroxyapatite, as alternative remineralization agents should be studied and randomized controlled trials comparing different fluoride concentrations and delivery methods could provide clearer guidelines for clinical application. Another area of interest is the role of artificial intelligence in monitoring oral hygiene compliance and detecting early signs of WSLs, which may enhance preventive strategies in orthodontic care.

For future RCTs, it is important to address several key factors, including sufficient sample size, independent allocation concealment, blinded assessments at both the patient and tooth levels, and the use of a validated core outcome set. Moreover, greater attention is needed to incorporate WSL-preventive technologies into routine orthodontic care. Further research is essential to establish clear and reproducible protocols for laser applications. Many studies have assessed the effectiveness of toothpaste and other products with antibacterial properties, with several showing promising results that warrant further exploration. Orthodontists must keep in mind that correcting malocclusions should go hand-in-hand with maintaining the patient’s oral and dental health, and it is hoped that caries prevention initiatives will continue to gain widespread attention and support.

## 5. Conclusions

In conclusion, WS is a common and equally dreaded complication of fixed orthodontics, as they risk seriously compromising the aesthetic and functional outcomes. The studies analyzed in this literature review have highlighted the importance of preventing white spot lesions, which occur during traditional fixed orthodontic therapy with brackets. In this context, the role of not only the orthodontist but also the dental hygienist is crucial for educating and motivating the orthodontic patient, who is often an adolescent and thus at a delicate stage in their development. To reduce the development of white spot lesions, it is essential to maintain optimal oral hygiene, complemented by high-fluoride toothpastes and mouthwashes as part of the patient’s daily routine. Sometimes, these measures alone may not be sufficient to guarantee optimal results. Orthodontic therapy aims to improve not only functionality and oral health but also the aesthetics of the smile, and the presence of white spot lesions could compromise the positive outcome of a long and demanding treatment. It is therefore essential to integrate other products, such as fluoride varnishes and casein phosphopeptide-amorphous calcium phosphate compounds, which have proven effective in preventing and remineralizing these lesions, helping to reduce their incidence. Orthodontists should consider integrating evidence-based preventive strategies into routine patient care, prioritizing passive fluoride applications for patients with low compliance. Personalized oral hygiene instruction, combined with the application of remineralizing agents, may offer a more comprehensive approach to minimizing the risk of WSLs. Future research should focus on optimizing treatment protocols, evaluating the long-term effectiveness of various interventions, and identifying strategies to improve patient adherence to preventive measures. While this review provides valuable insights into WSL prevention strategies, it is essential to acknowledge its limitations. The heterogeneity among studies in terms of methodologies and intervention protocols presents challenges in drawing definitive conclusions. Additionally, most included studies have short-term follow-up periods, making it difficult to assess whether remineralization effects are maintained after orthodontic treatment completion. Future research should aim to standardize study designs and implement long-term clinical trials to validate the efficacy of these preventive strategies. However, the literature still does not provide definitive evidence regarding the preventive capacity of different products, as there are studies with conflicting results on the prevention of WSLs.

WS prophylaxis begins with the correct choice and motivation of the subject to maintain good hygiene. In this regard, good oral hygiene with fluoride-containing toothpaste is the essential starting point for the effective removal of food scraps and bacterial biofilm that are deposited on teeth and braces. In addition, fluoride administration with mouthwashes for home use as well as gels, varnishes, and sealants for periodic professional use may be considered, depending on the case.

## Figures and Tables

**Table 1:** Summary of the literature on white spot lesion (WSL) prevention and treatment during fixed orthodontic treatment.

Title and Reference	Authors and Date	Objective	Materials and Methods	Conclusions
Prevention and Treatment of White Spot Lesions During and After Treatment with Fixed Orthodontic Appliances: a Systematic Literature Review [13]	Lopatiene K, Borisovaite M, Lapenaite E (2016)	Update evidence on the prevention of WSLs using fluoride and casein-based supplements.	Systematic review of controlled studies (2008–2016) on human subjects undergoing orthodontic treatment with fixed appliances. Searches conducted in PubMed, ScienceDirect, Embase, and Cochrane Library.	Fluoride and casein supplements effectively reduce WSLs. Casein phosphopeptide-amorphous calcium phosphate (CPP-ACP) may be more effective than fluoride rinses in reducing demineralization spots.
Fluorides for preventing early tooth decay (demineralised lesions) during fixed brace treatment [14]	Benson PE, Parkin N, Dyer F, Millett DT, Germain P (2019)	Evaluate the effectiveness of topical fluoride in reducing the percentage of new WSLs in orthodontic patients.	Randomized controlled trials comparing fluoride-containing products with placebos or no treatment. Searches conducted in Cochrane Library, Medline, and Embase.	High-fluoride toothpaste (5000 ppm) and professional application of fluoride foam (12,300 ppm) reduce new WSLs effectively during fixed orthodontic treatment.
Prevention of white spot lesions with fluoride varnish during orthodontic treatment with fixed appliances: a systematic review [15]	Sonesson M, Twetman S (2023)	Evaluate the preventive effect of fluoride varnish on WSLs during orthodontic treatment.	Systematic review of randomized clinical trials (up to 2022) with at least quarterly applications of fluoride varnish.	Fluoride varnish effectively prevents WSLs during orthodontic treatment when applied regularly.
Caries prevention during orthodontic treatment: In vivo assessment of high-fluoride varnish to prevent white spot lesions [16]	Perrini F, Lombardo L, Arreghini A, Medori S, Siciliani G (2016)	Evaluate the effectiveness of fluoride varnish in preventing WSLs in patients with fixed appliances.	Split-mouth study with 24 orthodontic patients, evaluating demineralization changes on varnished vs. non-varnished teeth.	Periodic fluoride varnish application offers some protection against WSLs but is not statistically significant with excellent oral hygiene.
Fluoride varnish for the prevention of white spot lesions during orthodontic treatment with fixed appliances: a randomized controlled trial [17]	Sonesson M, Brechter A, Abdulraheem S, Lindman R, Twetman S (2020)	Evaluate the effectiveness of ammonium fluoride varnish in preventing WSLs.	166 patients randomized to treatment or placebo groups; fluoride varnish applied around brackets every six weeks.	Ammonium fluoride varnish reduces advanced WSLs during orthodontic treatment.
An Extensive Comparison of the Clinical Efficiency of Acidulated Phosphate Fluoride (APF) and Neutral Sodium Fluoride (NaF) Oral Rinses in the Prevention of White Spot Lesions during Fixed Orthodontic Treatment: A Randomized Controlled Trial [18]	Pilli LN, Singaraju GS, Nettam V, Keerthipati T, Mandava P, Marya A (2022)	Compare weekly NaF mouthwash with daily APF mouthwash in reducing WSLs.	90 participants randomized into two groups for six months of intervention.	Daily APF mouthwash is more effective than weekly NaF in preventing WSLs.
Systematic review of preventive and treatment measures regarding orthodontically induced white spot lesions [19]	Yazarloo S, Arab S, Mirhashemi AH, Gholamrezayi E (2023)	Examine evidence-based measures for WSL prevention and treatment.	Systematic review of randomized clinical trials (2015–2020).	Brushing with fluoride toothpaste is foundational; 5% fluoride varnish is recommended for poor oral hygiene.
Effectiveness of self-applied topical fluorides against enamel white spot lesions from multi-bracketed fixed orthodontic treatment: a systematic review [20]	Sardana D, Manchanda S, Ekambaram M, Yang Y, McGrath CP, Yiu CKY (2019)	Evaluate self-applied fluorides in WSL prevention and recovery.	Systematic review of randomized controlled trials; searches in Cochrane Library, Embase, Medline, and Scopus.	Partial support for self-applied fluorides in WSL prevention; less evidence for recovery.
Efficacy of CPP-ACP and CPP-ACPF for Prevention and Remineralization of White Spot Lesions in Orthodontic Patients: a Systematic Review of Randomized Controlled Clinical Trials [21]	Imani MM, Safaei M, Afnaniesfandabad A, Moradpoor H, Sadeghi M, Golshah A, Sharifi R, Mozaffari HR (2019)	Evaluate CPP-ACP and CPP-ACPF effectiveness in WSL prevention and remineralization.	Systematic review of trials retrieved from Web of Science, Scopus, PubMed, and Cochrane Library.	CPP-ACP and CPP-ACPF reduce WSL prevalence and increase remineralization.
Effectiveness of amine fluoride mouthwash in preventing white spot lesions during fixed orthodontic therapy—A randomized control trial [22]	Ravi Kiran KR, Sabrish S, Mathew S, Shivamurthy PG, Sagarkar R (2023)	Evaluate the effect of amine fluoride mouthwash in preventing WSLs during fixed orthodontic therapy.	Randomized trial with 50 patients, divided into two groups. Analysis based on intraoral photographs assessing the percentage of tooth surface affected by WSLs.	Amine fluoride mouthwash significantly reduces WSLs compared to fluoride toothpaste alone.
Preventive Effect of Professional Fluoride Supplements on Enamel Demineralization in Patients Undergoing Fixed Orthodontic Treatment: A Systematic Review and Meta-Analysis [23]	Babadi Oregani E, Jafari A, Masoud Sajedi S, Reza Motamedian S (2022)	Review evidence on professional fluoride interventions in WSL prevention.	Systematic review of RCTs from PubMed and Cochrane Library, including studies on varnishes, gels, and mouthwashes.	Multiple applications of fluoride varnish or daily fluoride mouthwash significantly reduce WSLs.
MI Varnish and MI Paste Plus in a caries prevention and remineralization study: a randomized controlled trial [24]	Rechmann P, Bekmezian S, Rechmann BMT, Chaffee BW, Featherstone JDB (2018)	Evaluate the effect of MI Paste Plus (MIPP) and MI Varnish (MIV) on WSLs in orthodontic patients.	40 patients randomized into experimental (fluoride toothpaste, MIPP, MIV) and control groups. Data collected over 12 months.	Higher salivary fluoride in the experimental group, but no statistically significant differences in WSL scores.
Effectiveness of high-fluoride toothpaste on enamel demineralization during orthodontic treatment-a multicenter randomized controlled trial [25]	Sonesson M, Twetman S, Bondemark L (2014)	Evaluate high-fluoride toothpaste for preventing WSLs during orthodontic treatment.	424 adolescents randomized to use toothpaste with either 5000 ppm or 1450 ppm fluoride.	Daily use of high-fluoride toothpaste significantly reduces WSLs.
Evaluation of the Efficacy of Various Topical Fluorides on Enamel Demineralization Adjacent to Orthodontic Brackets: An In Vitro Study [26]	Reddy R, Manne R, Sekhar GC, Gupta S, Shivaram N, Nandalur KR (2019)	Compare different topical fluorides for preventing demineralization around brackets.	In vitro study on 100 premolars, divided into five groups. Test agents included fluoride varnish, APF gel, and fluoride toothpaste.	Fluoride varnish performed best, followed by fluoride toothpaste, APF gel, and mouthwash.
White Spots: Prevention in Orthodontics-Systematic Review of the Literature [27]	Patano A, Malcangi G, Sardano R, Mastrodonato A, Garofoli G, Mancini A, Inchingolo AD, Di Venere D, Inchingolo F, Dipalma G, Inchingolo AM (2023)	Identify effective strategies for preventing WSLs during orthodontic treatment.	Systematic review of articles published (2018–2023) on human subjects undergoing orthodontic treatment.	Good oral hygiene with fluoride toothpaste is essential; professional fluoride gels and varnishes are recommended for periodic application.
Effect of fluoride dentifrices on white spot lesions during orthodontic treatment: A randomized trial [28]	Kau CH, Wang J, Palombini A, Abou-Kheir N, Christou T (2019)	Assess the effect of Clinpro 5000, Clinpro Tooth Crème, and MI Paste Plus on WSLs during orthodontic treatment.	Randomized trial with three groups brushing with selected products for four months.	Clinpro 5000 is marginally more effective than Clinpro Tooth Crème and MI Paste Plus in reducing WSLs.
Prevention of white spot lesions using three remineralizing agents: An in vitro comparative study [29]	Tahmasbi S, Mousavi S, Behroozibakhsh M, Badiee M (2024)	Compare the effectiveness of NaF, CPP-ACPF, and Remin Pro in preventing demineralization.	56 premolars subjected to pH cycling with remineralizing agents applied daily.	NaF is more effective than CPP-ACPF and Remin Pro in preventing WSLs.

**Table 2:** Summary of the risk of bias for RCT studies according to the Cochrane Collaboration tool.

Authors and Reference	Design	Random Sequence Generation	Allocation Concealment	Blinding of Outcome Assessment	Incomplete Outcome Data	Selective Reporting	Risk of Bias
Benson et al. [14]	RCT	Low	High	Unclear	Low	Low	High
Perrini et al. [16]	RCT	Low	High	Unclear	Low	Low	High
Sonesson et al. [17]	RCT	Low	Low	Low	Low	Unclear	Moderate
Pilli et al. [18]	RCT	Low	High	Low	Unclear	Low	High
Ravi et al. [22]	RCT	Low	Low	Low	Low	Unclear	Moderate
Rechmann et al. [24]	RCT	Low	Low	Low	High	Unclear	High
Sonesson et al. [15]	RCT	Low	Low	Low	Low	Unclear	Moderate
Kau et al. [28]	RCT	Low	Low	Unclear	High	High	High

## Data Availability

Data available upon request to Corresponding Author.

## Appendix A

PubMed	(((white spot) OR (demineralized lesion) OR (demineralization)) AND ((fixed orthodontic treatment) OR (orthodontics) OR (orthodontic) OR (dental appliance) OR (orthodontic appliance)) AND ((CPP-ACP) OR (casein phosphopeptide) OR (amorphous calcium phosphate) OR (topical fluoride) OR (fluoride)))
EMBASE	#white spot* OR #white lesion* OR #demineralization OR #demineralized lesion* AND orthodontic* OR #orthodontia OR #fixed appliance* OR #dental orthopedics OR #dental orthopeadics AND #CPP-ACP OR #casein phosphopeptide OR #amorphous calcium phosphate OR #topical fluoride OR #fluride
Scopus	“white spot” OR “demineralized lesion” AND “orthodontics” OR “fixed orthodontic appliance OR “fixed orthodontic treatment” AND “CPP-ACP” OR “casein phosphopeptide OR “amorphous calcium phosphate” OR “topical fluoride” OR “fluride”
OpenGrey	orthodontics AND white spot AND topical fluoride
